# Calcium Channel Blocker Overdose With Cardiogenic Shock

**DOI:** 10.7759/cureus.109694

**Published:** 2026-05-26

**Authors:** Adedolapo V Adejumo

**Affiliations:** 1 Intensive Care Unit, Scunthorpe General Hospital, Northern Lincolnshire and Goole NHS Foundation Trust, Scunthorpe, GBR

**Keywords:** calcium channel blocker overdose, catecholamine-refractory shock, dihydropyridine calcium channel blocker, high-dose insulin euglycemic therapy, multi-organ failure

## Abstract

Calcium channel blocker (CCB) overdose is a rare but potentially life-threatening presentation associated with profound bradycardia, shock, and metabolic disturbances. Management can be complex, often requiring advanced hemodynamic support and multimodal therapies. We report a case of severe CCB intoxication leading to refractory hypotension, illustrating the challenges encountered in critical care management. Current approaches involve multimodal pharmacological interventions, including high-dose insulin euglycemic therapy, IV calcium, glucagon, and lipid rescue. CCBs have widespread use; therefore, understanding the consequences of overdose is clinically important.

## Introduction

Calcium channel blockers (CCBs) are widely used cardiovascular drugs that inhibit L-type voltage-gated calcium channels in cardiac and vascular smooth muscle, reducing intracellular calcium and thereby causing vasodilation and negative chronotropic and inotropic effects. Two major subclasses exist: dihydropyridines act predominantly on vascular smooth muscle, producing peripheral and coronary vasodilation with minimal direct cardiac depression, while nondihydropyridines are more cardiac selective, slowing sinoatrial and atrioventricular nodal conduction and decreasing contractility and heart rate [1].

Common indications, particularly in the United Kingdom, include hypertension, coronary artery disease, and supraventricular tachyarrhythmias. In a large UK primary care cohort (2.7 million patients, 1988-2018), CCBs were one of the two most frequently used antihypertensive classes [2]. Amlodipine is the dominant agent in routine practice, representing about 95% of all CCB prescriptions.

CCBs are recognized as a major contributor to drug-related fatalities among cardiovascular drugs because severe poisoning can cause profound hypotension, bradycardia, and cardiogenic shock [1]. In data from the Danish Poisons Information Center, 2% of patients with CCB exposures died within 30 days; all deaths occurred in adults with suicidal, mixed-drug overdoses [3]. Overall, in normal therapeutic use, CCBs have a favorable safety profile, but deliberate or massive overdose carries a low-incidence but high-severity mortality risk.

## Case presentation

We present the case of a 64-year-old female with a history of anxiety, depression, previous mixed overdose, and hypertension, who was found unresponsive. She was brought to the ED by paramedics due to suspected ingestion of a combination of medications, as empty medication containers of amlodipine and blister packs of other medications were found at the scene.

On arrival, she was maintaining her airway with an oropharyngeal airway. There was bilateral air entry with clear breath sounds, and oxygen saturation was 96% on supplemental oxygen delivered via a 15 L non-rebreather mask. She had a blood pressure of 60/40 mmHg with a heart rate of 80 beats per minute. The Glasgow Coma Score was 3/15, and she was not hypothermic. The initial ECG showed sinus rhythm, with no evidence of PR interval or corrected QT interval prolongation. The QRS interval was also normal. Arterial blood gas analysis was normal.

Resuscitation was initiated with IV fluids, and naloxone marginally improved the coma score to 8. Blood samples were sent for laboratory analysis, including paracetamol and salicylate levels, and a urine toxicology screen was performed for drugs of overdose. Urine toxicology was negative for opiates and benzodiazepines and positive for quetiapine and promethazine. Results of initial and further investigations are shown in Table 1.

**Table 1:** Trend of laboratory investigations

Test	On admission	Day 1	Day 2	Day 3	Day 4
White cell count (× 10⁹/L)	11.1	12.6	15	16.4	14.6
Neutrophil count (× 10⁹/L)	8.7	9.5	10.97	13.4	12.6
C-reactive protein (mg/L)	0.6	8.7	40	54	46
Creatinine (µmol/L)	77	84	100	65	65
Lactate (mmol/L)	2.9	3.5	2.3	3.6	3.2
Alanine transaminase (U/L)	12	119	255	373	143
Serum glucose (mmol/L)	5	6.3	10.1	11.2	10.7

A CT head performed in the ED ruled out an intracranial event.

Despite adequate fluid resuscitation, she remained profoundly hypotensive and was transferred to the intensive care unit. She arrived in the ICU on a peripheral vasopressor to maintain blood pressure, with ongoing fluid resuscitation and urine output monitoring. She underwent rapid sequence induction and intubation due to desaturation with escalating oxygen requirements and concern for aspiration. A post-intubation chest radiograph revealed bilateral basal opacities with partial collapse of the left lower lobe.

Central venous access and an arterial line were inserted for invasive hemodynamic monitoring. Norepinephrine was commenced; however, she remained persistently hypotensive despite escalating vasopressor support. The National Poison Information Service was contacted and recommended [3] initiating treatment with a glucagon bolus of 5-10 mg IV and high-dose insulin euglycemic therapy (HIET) with a bolus of 1 unit/kg followed by an infusion titrated up to 10 units/kg/hour, with concurrent 10% dextrose infusion to maintain glucose >10 mmol/L and ongoing potassium monitoring. IV calcium therapy was also commenced. An urgent echocardiogram showed an ejection fraction >55% with normal sizes of both ventricles.

**Figure 1:**
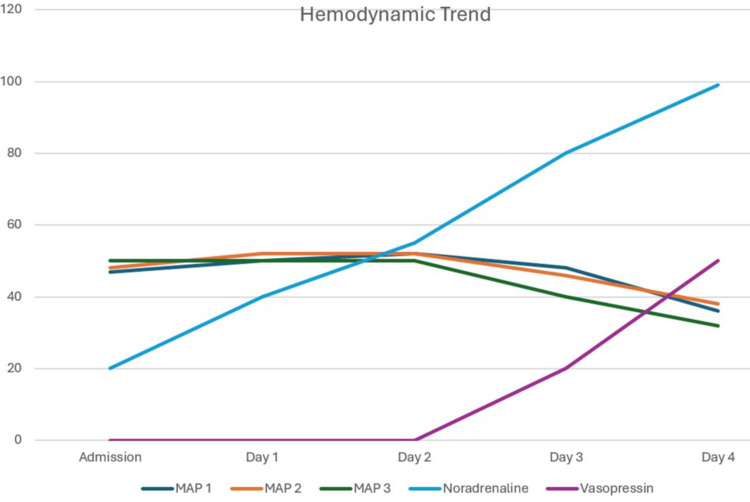
Trend of hemodynamics comparing mean arterial blood pressure to vasopressor requirements Noradrenaline (mcg/kg/hour); vasopressin (units/hour) MAP, mean arterial blood pressure

She remained profoundly hypotensive, requiring the addition of vasopressin to norepinephrine (Figure 1). Arterial blood gases showed significant acidemia consistent with combined metabolic and respiratory acidosis, as shown in Table 2. Renal replacement therapy was initiated, and muscle relaxants were administered to facilitate ventilation.

**Table 2:** Arterial blood gases during the course of care

Parameter	On admission	Day 1 (early)	Day 1 (late)	Day 2 (early)	Day 2 (late)	Day 3 (early)	Day 3 (late)	Day 4 (early)	Day 4 (late)
pH	7.23	7.18	7.27	7.27	7.19	7.21	7.08	7.07	7.1
pO₂ (kPa)	13.9	11.5	12.3	10.8	8.49	8.44	7.7	8.8	9.19
pCO₂ (kPa)	5.5	5.7	6.22	6.34	7.31	7.11	7.2	7.4	9.3
FiO₂ (%)	60	60	60	60	50	50	70	70	80
Lactate	2.9	3.5	1.9	1.4	2.3	2.2	3.6	2.7	3.2
Bicarbonate (mmol/L)	17.2	16.3	21.4	21.8	21.2	21.5	19.5	19.8	22.1
Base excess (mmol/L)	-10	-11.5	-5.6	-5.1	-7	-6.4	-10.4	-10.5	-7.8

Over the following 72 hours, her clinical state continued to worsen despite maximum doses of HIET, dual vasopressor support, worsening mean arterial blood pressure, increasing oxygen requirements, and evolution of mixed acidosis not responding to continuous hemofiltration. She passed away due to multi-organ failure.

## Discussion

CCBs are among the most lethal cardiovascular drugs in overdose. Their mechanism of toxicity includes inhibition of L-type calcium channels within the myocardium, vascular smooth muscle, and pancreatic beta cells.

CCB overdose can present with a profound toxidrome of bradyarrhythmia, hypotension, and metabolic derangements. While non-dihydropyridine CCBs are typically associated with direct cardiac effects and dihydropyridines with vascular effects, this distinction may be lost in high-dose ingestions, and both features often occur. Cardiac effects include bradyarrhythmias such as atrioventricular block, escape rhythms, and asystole. Hyperglycemia is common due to impaired insulin release from pancreatic beta cells.

CCB overdose represents one of the most challenging toxicological presentations in critical care. Sustained-release formulations potentiate toxicity due to delayed absorption, often necessitating prolonged therapeutic interventions. Pharmacokinetic variability, including large volume of distribution, high protein binding, and ionization at physiological pH, contributes to prolonged toxicity.

Conventional therapies often prove ineffective in severe cases. Calcium infusion typically yields only a transient benefit. HIET has emerged as a mainstay of treatment, improving myocardial function and cellular metabolism while reversing hyperglycemia. Survival is improved when HIET is initiated early [2].

Lipid emulsion therapy may be considered for highly lipid-soluble agents such as verapamil, although evidence is inconsistent. Refractory cases may require mechanical circulatory support, including extracorporeal membrane oxygenation [4]. This highlights the importance of early toxicology consultation and consideration of extracorporeal techniques in severe CCB toxicity with refractory shock.

Hemodialysis is ineffective for CCB removal due to high protein binding, lipophilicity, and hepatic metabolism. Levosimendan has been reported to improve calcium sensitivity in overdose, but evidence remains limited [5]. Glucose levels correlate with the severity of CCB intoxication, and profound hyperglycemia is a marker of severe toxicity [6].

## Conclusions

CCB toxicity is frequently severe and often refractory to standard interventions. Management requires early intensive care and toxicology involvement, prompt initiation of HIET, and consideration of multimodal therapies including calcium, glucagon, and lipid rescue. Extracorporeal membrane oxygenation may improve outcomes. Despite aggressive management, mortality remains high in severe cases of shock.
